# The effect of China’s many-child policy on the number of births and the prevalence of serious teratogenic and disabling defects in Hunan Province

**DOI:** 10.1186/s12889-023-16583-x

**Published:** 2023-11-11

**Authors:** Donghua Xie, Jianhui Wei, Aihua Wang, Lili Xiong, Kehan Zou, Zhiqun Xie, Junqun Fang

**Affiliations:** 1 Department of Information Management, Maternal and Child Health Hospital of Hunan Province, 58 Xiangchun Road, Changsha, Hunan 410078 China; 2 NHC Key Laboratory of Birth Defect for Research and Prevention (Hunan Provincial Maternal and Child Health Care Hospital), 58 Xiangchun Road, Changsha, Hunan 410078 China; 3 Xiangya School of Public Health, Central South University, Changsha, China

**Keywords:** Many-child policies, Prevalence of serious teratogenic and disabling defects, Number of births

## Abstract

**Background:**

To research the effect of China’s many-child policy on the number of births and the prevalence of serious teratogenic and disabling defects (STDDs) in Hunan province.

**Methods:**

We performed an observational study based on the Birth Defect (BD) Surveillance System of Hunan Province and chose STDD case cards. From 2012–2022, we defined the following 4 periods: the one-child policy (OCP) (2012.01–2013.12), partial two-child policy (PTCP) (2014.1–2015.12), universal two-child policy (UTCP) (2016.1–2020.12), and the early stage of the three-child policy (ETCP) (2021.1–2022.12). Crude odds ratios (ORs) and 95% confidence intervals (CIs) were calculated to examine the association of policy changes with STDDs. *Crame′rʹs* V was calculated to estimate the effect sizes. Joinpoint regression analysis and annual percent change (APC) were used for each segment of the trend.

**Results:**

A total of 1,652,079 births were included in this analysis. Joinpoint regression analysis showed that the number of perinatal births increased from 2012 to 2017, with APC = 9.52 (95% CI: 7.2 to 11.8), and decreased from 2017 to 2022, with an APC = -10.04 (95% CI: -11.9 to -8.1). The number of mothers over 30 years old gradually increased, from 25.54% during the OCP period to 54.05% during the ETCP period (*P*_*trend*_ < 0.001).

With policy changes, the total prevalence of STDDs increased from 28.10 per 10,000 births during the period of OCP into 46.77 per 10,000 births during the ETCP period by 66.44%. The live birth prevalence of STDDs increased only during the ETCP period (PTCP: OR = 1.27, 95% CI: 0.99–1.24, *p* = 0.057, UTCP: OR = 1.22, 95% CI: 0.99–1.52, *p* = 0.067, ETCP: OR = 1.75, 95% CI: 1.37–2.24, *p* < 0.001). Over the past ten years, there was a decrease in the gestational age at diagnosis (*F = 772.520, *p* < 0.001), from 24.49 ± 5.65 weeks in 2012 to 20.77 ± 5.17 weeks in 2022. From 2012 to 2022, the percentage of deaths within 7 days decreased with APC = -18.85 (95% CI: -26.4— -10.5, *P* > 0.05).

**Conclusion:**

Many-child policies were associated with a moderate increase in fertility especially for women in urban areas and older women. However, they have lost the ability to control birth since 2017. The total prevalence of STDDs increased over the entire period, but the live birth prevalence increased only during the ETCP period. The gestational age at diagnosis decreased and the percentage of deaths within 7 days decreased.

## Introduction

China has the largest population in the world, affecting the country’s development [1]. In order to control population growth, the Chinese government implemented the one-child policy (OCP) in 1979 [2]. Thereafter, China’s birth rate dropped rapidly and many social problems appeared, including high sex ratio, a labor force shortage, population aging and so on [3–6]. In November 2013, the Chinese government declared a partial two-child policy (PTCP), allowing married couples to have a second child if either parent was a singleton [7]. In October 2015, the Chinese government implemented universal two-child policy (UTCP) [5]. To improve the population structure and actively address the aging population, on May 31, 2021, China implemented the three-child policy to allow each couple to have up to three children [8, 9]. With the expansion of the multiple-child policy, many women who did not intend to have a second or third child have tried to conceive. Therefore, these women face many challenges, including older age, second cesarean section, and neonatal health problems [10–12]. Among neonatal health problems, birth defects (BDs) are an important issue [10].

Some studies have shown that after the implementation of the two-child policy, the mean age of pregnant women increased significantly compared with that before the policy [13]. Previous studies have indicated that pregnant women aged ≥ 35 years have a higher risk of carrying a malformed fetus (OR = 1.24) [14]. A study by Xiaohui Zhang showed that the total prevalence of BDs in 2013, 2015, and 2017 was 245.95, 264.86, and 304.36 per 10,000 births, respectively, and that the live birth rate for infants with BDs born before 28 gestational weeks increased from 1.29% to 11.45% [15]. At present, some issues need to be addressed. First, there is the question of how the government’s three-child policy will change the population and the rate of BDs. Second, with the change in medical technology, minor defects can be corrected quickly, but there is little research on prenatal diagnosis abilities, medical intervention and prognosis of serious teratogenic and disabling defects (STDDs).

Until 2020, there was no definition of STDDs. The China Health Commission (CHC) published the types of STDDs and required using the prevalence of STDDs to be used as the main index of BD monitoring. Our study aimed to research the effect of China’s many-child policy on the number of births and the prevalence of STDDs.

## Methods

### Study design

We performed an observational study based on the BD Surveillance System of Hunan Province, which involves 52 hospitals with a uniform distribution throughout Hunan Province. From this system, we collected data between 2012.01.01 and 2022.12.31. The data included case cards for every child with BDs and a quarterly table of the number of perinatal infant numbers for every registered hospital. The termination of all pregnancies (TOP) affected by BDs, regardless of gestational age, was reported. From all BDs, we chose STDD cards. To fully consider the time interval between conception and delivery, we defined four periods: the OCP (births from January 2012 to December 2013), the PTCP (births from January 2014 to December 2015), the UTCP (births from January 2016 to December 2020), and the early stage of the three-child policy (ETCP) (births from January 2021 to December 2022).

### Patient and public involvement statement

This surveillance program was initiated by the National Health Commission which required all the surveillance hospitals to report BDs cards once happened in the admission office. The information on this card were from case information and patient’s presentation. Doctors signed informed consent from the patients after telling them to report the BD for analysis to determine the cause. This study was approved by the Ethics Committee of Maternal and Child Care, Hospital of Hunan Province. All personal information was removed before the data were analyzed.

### Monitoring method

According to the Maternal and Child Health Monitoring program in China (MCHMPC) created by the National Health System, cases of STDDs were identified in the obstetrics departments and the neonatal departments. The exported data included the case card for every fetus or child with BDs and a quarterly table of the number of cases for every registered hospital. These case cards were completed by gynecological, pediatric, or neonatal doctors. The case card and the summary table of cases were reported quarterly, both on paper and online. They were audited step by step by the maternal and child health hospitals and health administrative departments, respectively. All staff who collected data and the doctors who diagnosed or filed STDD cards tried their best to guarantee the quality of the data monitoring, and they received strict step-by-step training. Periodic quality-control inspections and audits were conducted every quarter at the county level and twice per-year at the city level and the provincial level to reduce errors or missed data.

### Monitoring data

Every case card for STDDs included 5 components: maternal information (such as the mother’s residence and education level), infant birth information, STDD diagnoses, maternal illnesses and drug use during pregnancy, and a detailed family history of disease. The quarterly table of perinatal infant numbers included three months of detailed data for both the town and village of the surveillance hospital. For example,the number of perinatal infants for every maternal age group, the number of stillbirths, the number of newborn babies who died within 7 days, the number of BDs, and others.

### Diagnostic criteria for STDDs

The diagnosis of STDDs was based on the “Maternal and Child Health Monitoring Manual in China” provided by the MCHMSC. Experts from each monitoring hospital were responsible for confirming the STDDs and providing technical support for the hospitals regarding STDDs. According to the interpretation of STDDs by the CHC, STDDs were categorized as follows: neural tube defects (NTDs), Down syndrome (DS), gastroschisis, exomphalos, limb shortening, transposition of the great arteries (TGA), tetralogy of Fallot (TOF), and atrioventricular septal defect (AVSD).

### Statistical analysis

Microsoft Excel 2010 was used for preliminary data analysis. SPSS 21.0 was used for the descriptive analyses. Piecewise linear plots were drawn to show changes in the numbers of births and TOPs from 2012 to 2021.Crude odds ratios (ORs) and 95% confidence intervals (CIs) were calculated by R version 4.0.2 to examine the association of policy changes with the risk of STDDs, with the one-child policy period defined as the reference group. Analysis of variance (ANOVA) was used to test the association of gestational age at diagnosis with policy changes. *Crame′rʹs* V was calculated with the chi-squared test and chi-square trend test to estimate effect sizes. Joinpoint 4.8.0.1 was used to perform joinpoint regression analysis and calculate the annual percent change (APC) for each segment of the trend (equal to the year-over-year change in the rate, under the assumption that this change was constant for a segment). GraphPad Prism 8 was used to draw a forest plot of the ORs and 95% CIs. *P* < 0.05 was considered indicative of statistical significance.

The prevalence of STDDs included the total birth rate and live birth rate of infants with STDDs. The total birth prevalence of STDDs referred to the overall occurrence with the total number of STDDs as the numerator and the total number of births as the denominator. The live birth prevalence of STDDs indicated births after medical intervention, with the number of live births with STDDs as the numerator and the total number of births as the denominator.

## Results

### The impact of China’s many-child policy on the number and characteristics of births

**Fig. 1:**
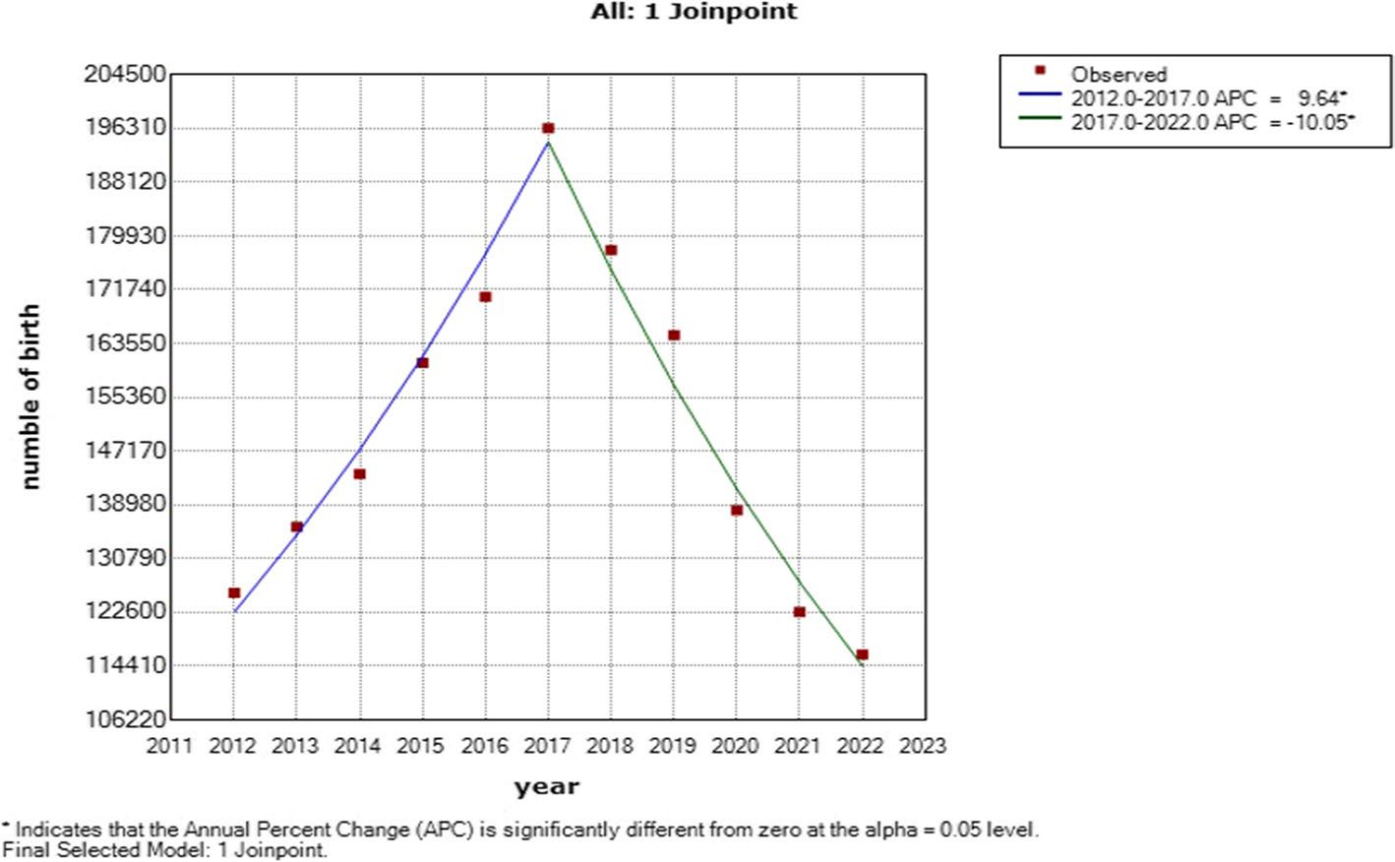
Joinpoint regression analysis of the trends in the number of births in Hunan Province from 2012 to 2021

A total of 1,535,901 births were included in this analysis. Joinpoint regression analysis showed that the number of perinatal births increased from 2012 to 2017, with an APC = 9.52 (95% CI: 7.2 to 11.8), and decreased from 2017 to 2022, with an APC = -10.04 (95% CI: -11.9 to -8.1) (Fig. 1).

According to the chi-square test, the correlations of maternal age, urban or rural area, fetal sex, and the number of fetus with policy change were significant (*P* < 0.05). With the implementation of the many-child policy, the number of pregnant women over 30 years old gradually increased from 25.54% during the OCP period to 55.07% during the ETCP period (*P*_*trend*_ < 0.001). Urban birth increased only during the PTCP period (from 44.49% to 47.05%) and showed a downward trend during other periods (*P* < 0.05). In comparison to the OCP period, the proportions of male births (from 52.95% to 53.16%, *p* = 0.037) and multiple births (from 2.26% to 2.96%, *P*_*trend*_ < 0.001) increased during the ETCP period (Table 1).

**Table 1:** Changes in birth characteristics over the period of policy changes

**Variables**	One-child policy period		Partial two-child policy period		Universal two-child policy period		Early stage of the three-child policy		Cramer’s V	*P*
	N	percent	N	percent	N	percent	N	percent		
Total births	261,228	100.00	304,269	100.00	847,755	100.00	238,827	100.00	——	——
**Maternal age**									0.153	< 0.001(Trend)
< 20	4560	1.75	6025	1.98	13,711	1.62	1790	1.37		
20–25	80,576	30.84	68,442	22.49	118,531	13.98	15,783	12.19		
25–30	109,371	41.87	143,841	47.27	357,581	42.18	38,774	31.37		
30–35	47,283	18.10	60,975	20.04	243,649	28.74	48,265	39.14		
≥ 35	19,438	7.44	24,986	8.21	114,283	13.48	18,037	15.93		
**Maternal region**									0.055	< 0.001
Urban	116,217	44.49	143,145	47.05	342,177	40.36	49,732	40.84		
Rural	145,011	55.51	161,124	52.95	505,578	59.64	72,917	59.16		
**Infant sex**									0.003	0.037
Male	138,309	52.95	161,019	52.92	448,287	52.88	65,210	53.16		
Female	122,867	47.03	143,204	47.06	399,368	47.11	57,422	46.83		
Unknown	52	0.02	46	0.02	100	0.01	17	0.01		
**Number of embryos**									0.028	< 0.001(Trend)
Single birth	255,317	97.74	296,392	97.41	818,986	96.61	119,031	97.04		
Multiple birth	5911	2.26	7877	2.59	28,769	3.39	3618	2.96		

*P* values and *Crame′rʹs* V were derived from chi-squared tests. *P*_trend_ was derived from trend analyses.

### The impact of China’s many-child policy on the prevalence of STDD

**Fig. 2:**
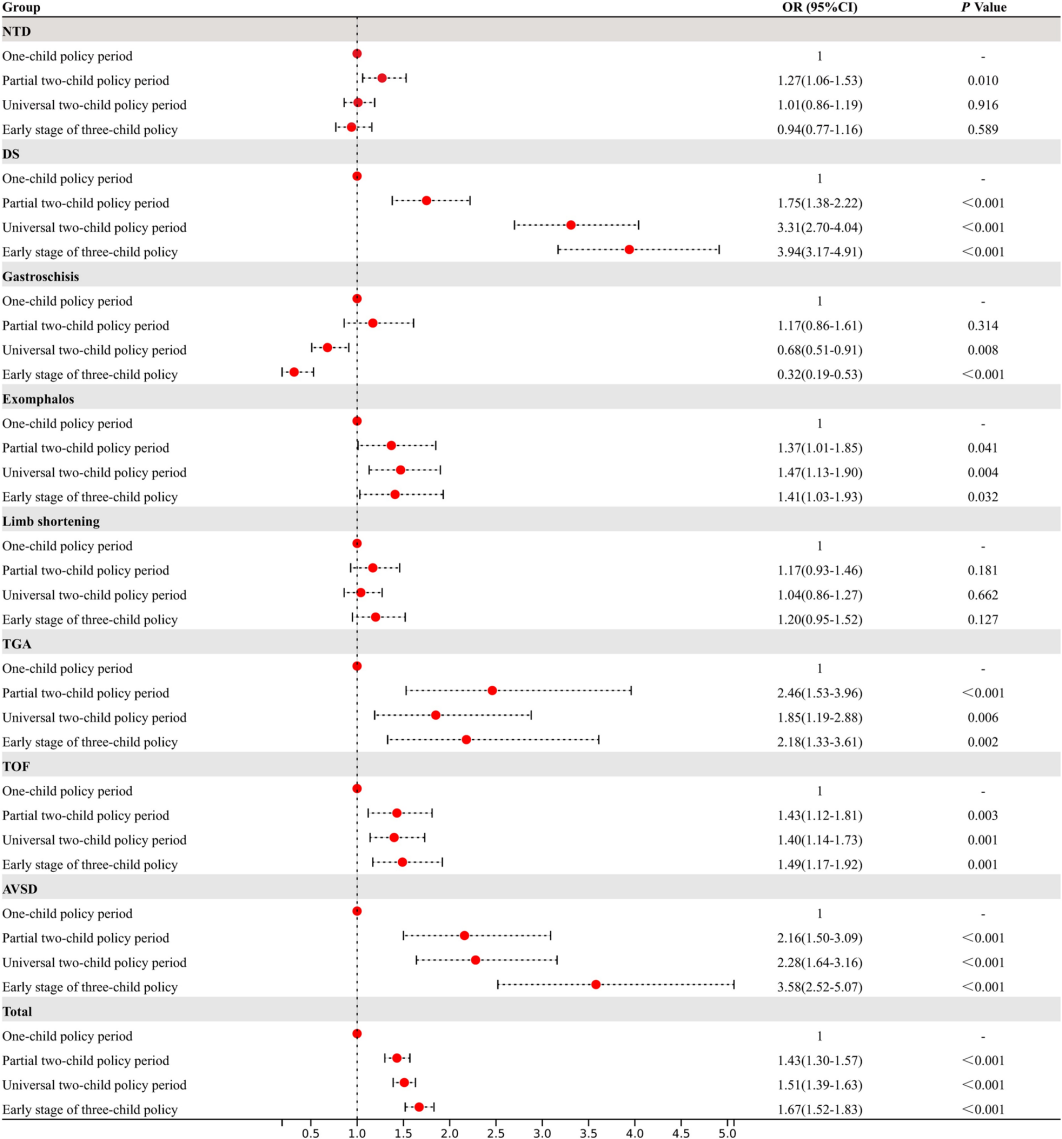
Crude ORs (95% CI) and* P* values for the association between birth policy changes and total prevalence of STDDs subtypes

With the policy changes, the total birth prevalence of STDDs increased from 28.10 per 10,000 births in the OCP period to 46.77 per 10,000 births in the ETCP period, an increase of 66.44% (Table 2). The total birth prevalence of STDDs and DS, TGA, TOF, and AVSD increased as follows: STDDs (PTCP: OR = 1.43, 95% CI: 1.31–1.57, *p* < 0.001, UTCP: OR = 1.51, 95% CI: 1.39–1.63, *p* < 0.001, ETCP: OR = 1.67, 95% CI: 1.52–1.83, *p* < 0.001), DS (PTCP: OR = 1.75, 95% CI: 1.38–2.22, *p* < 0.001, UTCP: OR = 3.31, 95% CI: 2.70–4.04, *p* < 0.001, ETCP: OR = 3.94, 95% CI: 3.17–4.91, *p* < 0.001), TGA (PTCP: OR = 2.46, 95% CI: 1.53–3.96, *p* < 0.001, UTCP: OR = 1.85, 95% CI: 1.19–2.88, *p* < 0.001, ETCP: OR = 2.18, 95% CI: 1.33–3.61, *p* < 0.001), TOF (PTCP: OR = 1.43, 95% CI: 1.12–1.81, *p* < 0.001, PTCP: OR = 1.40, 95% CI: 1.14–1.73, *p* < 0.001, PTCP: OR = 1.49, 95% CI:1.17–1.92, *p* < 0.001), AVSD (PTCP: OR = 2.16, 95% CI:1.5–3.1, *p* < 0.001, UTP: OR = 2.28, 95% CI: 1.64–3.16, *p* < 0.001, ETCP: OR = 3.58 95% CI: 2.52–5.07, *p* < 0.001). Increasing trends of the prevalence of NTDs (*P*_utcp_ = 0.916, *P*_ETCP_ = 0.524), gastroschisis (*P*_ptcp_ = 0.314) and limb shortening (*P*_ptcp_ = 0.181, *P*_utcp_ = 0.662) were not obvious (Table 2 and Fig. 2).

**Table 2:** Changes in the prevalence of STDDs over the period of policy changes

STDD subtypes	One-child policy period				Partial two-child policy period				Universal two-child policy period				stage of three-child policy period			
	Totalbirth		Live birth		Total		live		Total		live		Total		live	
	N	Prevalence (per 10000births)	N	Prevalence (per 10000births)	N	Prevalence (per 10000births)	N	Prevalence (per 10000births)	N	Prevalence (per 10000births)	N	Prevalence (per 10000births)	N	Prevalence (per 10000births)	N	Prevalence (per 10000births)
NTD	190	7.27	5	0.19	282	9.27	18	0.60	622	7.34	44	0.53	164	6.87	15	0.49
DS	103	3.94	14	0.54	210	6.90	17	0.57	1103	13.01	45	0.54	371	15.53	12	0.51
gastroschisis	68	2.60	8	0.31	93	3.06	11	0.37	150	1.77	11	0.13	20	0.84	1	0.04
Exomphalos	69	2.64	14	0.54	110	3.62	11	0.37	328	3.87	58	0.69	89	3.73	13	0.55
limb shortening	131	5.01	37	1.43	178	5.85	62	2.06	444	5.24	119	1.42	144	6.03	46	1.94
TGA	23	0.88	2	0.08	66	2.17	7	0.23	138	1.63	17	0.20	46	1.93	8	0.34
TOF	109	4.17	14	0.54	181	5.95	13	0.43	496	5.85	53	0.63	149	6.24	39	1.64
AVSD	41	1.57	9	0.35	103	3.39	14	0.47	303	3.57	62	0.74	134	5.61	32	1.35
Total	734	28.10	103	3.99	1223	40.19	153	5.09	3584	42.28	409	4.89	1117	46.77	166	7.00

**Fig. 3:**
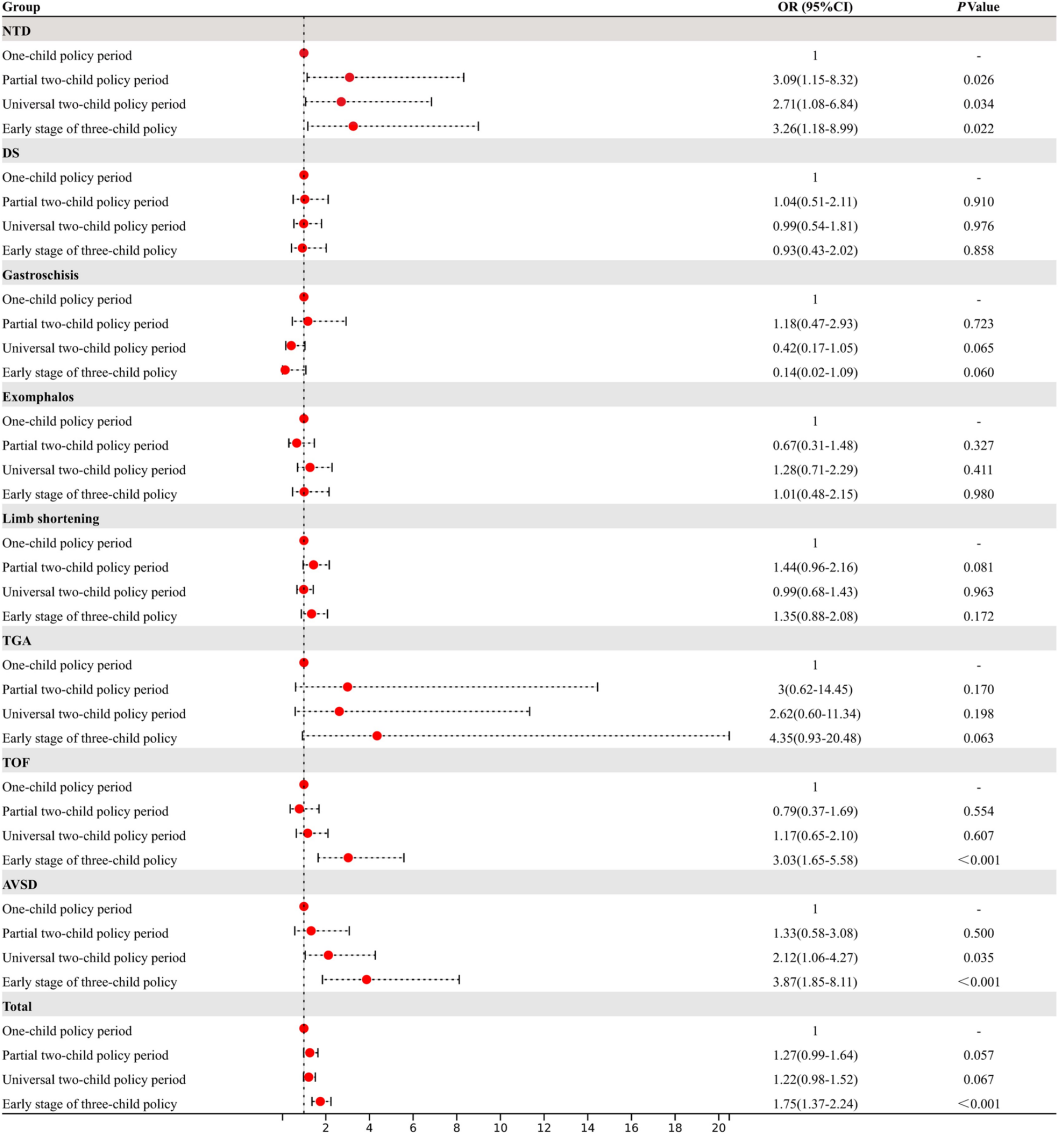
Crude ORs (95% CI) and* P* values for the association between birth policy changes and live prevalence of STDD subtypes

With the implementation of the many-child policy, the live birth prevalence of STDDs increased only during the ETCP period (PTCP: OR = 1.27, 95% CI: 0.99–1.24, *p* = 0.057, UTCP: OR = 1.22, 95% CI: 0.99–1.52, *p* = 0.067, ETCP: OR = 1.75, 95% CI: 1.37–2.24, *p* < 0.001). Among the subtypes, the live birth prevalence of NTDs increased, compared with the OCP period (PTCP: OR = 3.09, 95% CI: 1.15–8.32, *p* = 0.026, UTCP: OR = 2.71, 95% CI: 1.08–6.84, *p* = 0.034, ETCP: OR = 3.26, 95% CI: 1.18–8.99, *p* < 0.001). During the ETCP period, the live birth prevalence of NTD (ETCP: OR = 3.26, 95% CI: 1.18–8.98, *p* < 0.05), TOF (ETCP: OR = 3.03, 95% CI: 1.65–5.58, *p* < 0.001) and AVSD (ETCP: OR = 3.87, 95% CI: 1.85–8.11, *p* < 0.001) increased. Changes in other subtypes were not statistically significant from the OCP to ETCP periods (Table 2 and Fig. 3).

### The impact of China’s many-child policy on the diagnosis and outcome of STDDs

Over the past ten years, the gestational age at diagnosis decreased (*F = 772.520, *p* = 0.000) from 24.49 ± 5.65 weeks in 2012 to 20.77 ± 5.17 weeks in 2022, by reducing 3.72 weeks (Table 3).

**Table 3:** Changes in the diagnosis and outcome of STDDs over the period of policy changes

Years	Total STDD (n)	live STDD (n)	Prenatal diagnosis (n)	gestational age of diagnosis *(weeks)	TOP (n)	deaths within 7 days (n)	TOP(%)	Deaths within 7 days(%)
2012	355	60	301	24.49 ± 5.65	288	9	95.68	15.00
2013	379	43	338	24.20 ± 5.94	331	8	97.93	18.60
2014	530	79	465	23.59 ± 5.96	445	9	95.70	11.39
2015	693	74	632	23.47 ± 5.96	607	4	96.04	5.41
2016	787	92	709	22.82 ± 5.62	685	4	96.61	4.35
2017	721	83	656	22.19 ± 5.23	631	3	96.19	3.61
2018	715	97	637	21.98 ± 5.47	613	7	96.23	7.22
2019	731	64	675	21.34 ± 5.27	655	4	97.04	6.25
2020	630	73	565	20.86 ± 5.24	554	1	98.05	1.37
2021	561	77	498	21.12 ± 5.36	481	2	96.59	2.60
2022	556	90	501	20.77 ± 5.17	474	2	94.61	2.22
total	6658	832	5977	22.28 ± 5.63	5764	53	96.44	6.37

^*^F = 780.310, *p* = 0.000

**Fig. 4:**
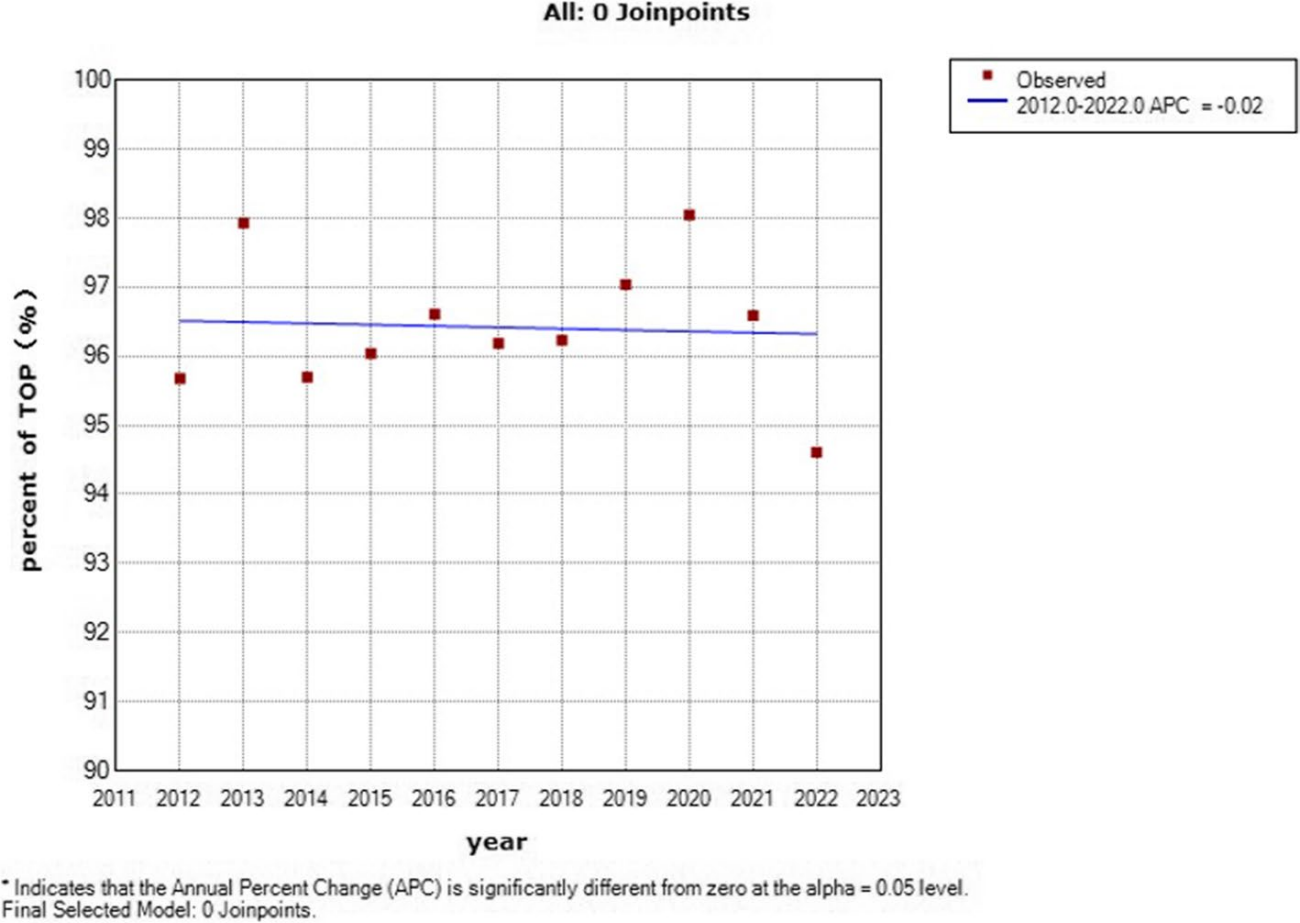
Joinpoint regression analysis of the trends of percent of TOP for STDDs: 2012–2021

**Fig. 5:**
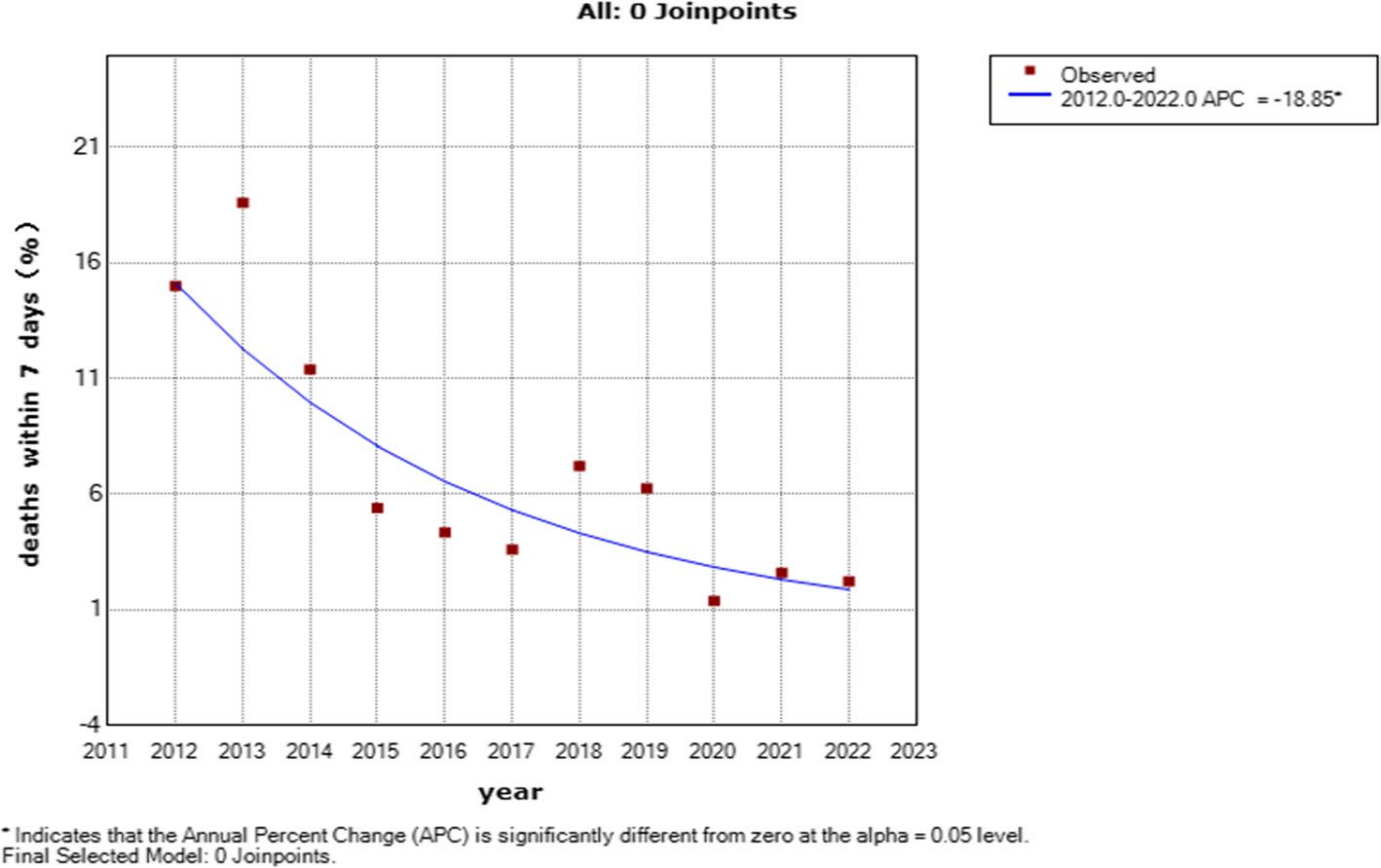
Joinpoint regression analysis of the trends of percent of deaths within 7 days: 2012–2021

From 2012 to 2022, there was no significant difference in the rate of termination of pregnancy (TOP), with an APC = -0.02 (95% CI: -0.3–0.2, *P* > 0.05) (Fig. 4). The percentage of deaths within 7 days decreased with an APC = -18.85 (95% CI: -26.4— -10.5, *P* < 0.05) (Fig. 5).

## Discussion

Our research showed the many-child policies had some effect on birth up to but not after 2017 in Hunan province. The data showed that the number of births increased from 2012 to 2017, with an APC = 9.52 (95% CI: 7.2 to 11.8), and decreased from 2017 to 2022, with an APC = -10.04 (95% CI: -11.9 to -8.1), which was similar to previous research [16, 17]. A study by Zhao Y showed that the number of births peaked in 2017, with yearbook and surveillance data showing increases of 15.92% and 31.15%, respectively, over 2014 [18]. The National Statistics Bureau reported the number of annual births as10.62 million for 2021, a sharp decline of 11.50 percent compared to the 12 million births reported in China’s 2020 census, and the fertility rate in China increased only in 2014 and 2016 [19]. It is possible that there were several reasons for this phenomenon. First, the number of women of childbearing age continues to decrease. In 2021, there will be approximately 5 million fewer women of reproductive age (between 15 and 49 years) than in 2020, including approximately 3 million fewer women between 21 and 35 years. Second, the fertility rate continued to decline. The total fertility rate of women of childbearing age continued to decline in 2021 due to the changing conception methods and the delay in the age of first marriage and childbirth (delayed by approximately 2 years over the past 10 years) [20]. Therefore, the family planning model and the many-child policies have gradually lost the power to affect the birth population after 2017 [4].

During the OCP period, couples were permitted to have 2 children in rural areas if the first child was a girl, but the OCP was strictly enforced in urban areas [7]. From the perspective of the people’s needs, the OCP produced many legacy problems, including a number of urban couples wanted to have a second child but could not, and couples wanted to have a boy but could not. Therefore, when the PTCP was enacted in November 2013, the number of births increased, especially for urban and older women [3, 13, 15, 21]. The male birth rates increased when the first child was female [21]. Due to older age and insufficient fertility, the number of babies conceived via in vitro fertilization increased, and the proportion of multiple births has increased [22, 23]. However, this phenomenon lasted only a short time, affecting only a few families in urban areas who were eager to have a second child. Most young parents abandoned the idea of having a second child, which was related to the family’s socioeconomic status because of the cost of childbearing and educational and occupational pressures, especially in urban areas [24]. Therefore, for the above reasons, our study showed that the percentages of mothers over 30 years old, male births, rural births, and multiple births gradually increased with the implementation of the many-child policy.

Many studies have shown that the many-child policies are associated with an increasing in maternal age [3, 23]. A study by Hanyi Chen showed that the proportion of mothers aged≧35 years increased from 7.39% during the OCP period to 10.59% during the PTCP period and to 13.65% during the UNCP period [3], which was similar to our study, increasing from 7.44% to 8.21%, 13.48%, and 14.71% across the four different periods. One meta-analysis showed that the pooled unadjusted ORs (95% CI) of total congenital anomalies were 1.64 (1.40–1.92) and 1.05 (0.95–1.15), and those of chromosomal anomalies were 5.64 (5.13–6.20) and 0.69 (0.54–0.88) in the older and younger age groups, respectively [25]. Wang R showed that the number of cases of chromosomal abnormalities increased in northeastern China in the past 2 years after the implementation of the two-child policy [26]. A study by Olukemi O Ige showed that in the severe congenital heart defect (CHD) group, maternal age (33.1 ± 5.1 years) was older than that in the mild CHD group (30.5 ± 5.7 years [27]. Preliminary data from the state vital statistics office showed a higher overall prevalence of BDs among older mothers [28]. In addition, with increased awareness of prenatal diagnosis, STDDs are more likely to be detected [29]. Therefore, our study revealed that the total prevalence of STDDs, DS, TGA, TOF, and AVSD increased by 66.45%, 293.97%, 118.76%, 49.52%, and 257.49%, respectively, from the OCP period to the ETCP period.

Additionally, the field of fetal cardiology has advanced regarding accurate prenatal diagnosis of severe CHD [30]. Moreover, the Hunan Provincial Government approved BD prevention and control and spent six million RMB to implement the integrated prenatal and postnatal prevention and treatment model for CHDs in 2021. Many fetuses with CHD, including TGA, TOF, and AVSD, were chosen to receive treatment after birth. In addition, the mortality rate of CHD surgery in children decreased yearly, which was similar to our study, with the percentage of deaths within 7 days decreasing, with an APC = -19.84 [31]. Therefore, with the implementation of many-child policies, the live birth prevalence of STDDs increased only during the ETCP period. The main cause was the increasing prevalence of live births affected by severe CHDs, including TGA, TOF, and AVSD. Another reason may be the considerable advances in the diagnosis and treatment of spina bifida, an NTD [32].

In recent years, there have been many advances in prenatal testing, including ultrasonography, analysis of serum markers, and amniotic fluid and chorionic villus sampling. On the one hand, three- and four-dimensional ultrasound, harmonic imaging, high-contrast resolution, speckle reduction, and one-touch images have been used increasingly earlier [33]. On the other hand, genetic testing technology, such as noninvasive prenatal testing (NIPT) using next-generation sequencing (NGS) of cell-free fetal DNA (cffDNA) followed by bioinformatic analysis, has kept skills current [34]. This technology is gaining acceptance in clinical practice [35]. Therefore, over the past ten years, the gestational age at diagnosis decreased in our study.

To date, there have been a few studies on the impact of the two-child policy on live births and birth defects, but no analysis has assessed serious defects, and the impact of the three-child policy is unknown. This study was conducted to illustrate the impact of many-child policies on births and STDDs from the OCP period to the ETCP period based on a large sample. However, 2021 -2022 was the initial period of the three-child policy, and the full impact of the three-child policy may not be shown. Therefore, we will continue to monitor and analyze the impact of the three-child policy.

## Data Availability

The datasets used and analyzed during the current study are available from the corresponding author on reasonable request.
